# Temperature-Mediated Effects on Mayaro Virus Vector Competency of Florida *Aedes aegypti* Mosquito Vectors

**DOI:** 10.3390/v14050880

**Published:** 2022-04-23

**Authors:** Abdullah A. Alomar, Barry W. Alto

**Affiliations:** Florida Medical Entomology Laboratory, Entomology and Nematology Department, Institute of Food and Agricultural Sciences, University of Florida, 200 9th St. S.E., Vero Beach, FL 32962, USA; bwalto@ufl.edu

**Keywords:** per os infection, arbovirus, climate, Mayaro, *Aedes aegypti*

## Abstract

Mayaro virus (MAYV) is an emerging mosquito-borne arbovirus and public health concern. We evaluated the influence of temperature on *Aedes aegypti* responses to MAYV oral infection and transmission at two constant temperatures (20 °C and 30 °C). Infection of mosquito tissues (bodies and legs) and salivary secretions with MAYV was determined at 3, 9, 15, 21, and 27 days post ingestion. At both temperatures, we observed a trend of increase in progression of MAYV infection and replication kinetics over time, followed by a decline during later periods. Peaks of MAYV infection, titer, and dissemination from the midgut were detected at 15 and 21 days post ingestion at 30 °C and 20 °C, respectively. Mosquitoes were able to transmit MAYV as early as day 3 at 30 °C, but MAYV was not detectable in salivary secretions until day 15 at 20 °C. Low rates of MAYV in salivary secretions collected from infected mosquitoes provided evidence supporting the notion that a substantial salivary gland barrier(s) in Florida *Ae. aegypti* can limit the risk of MAYV transmission. Our results provide insights into the effects of temperature and time on the progression of infection and replication of MAYV in *Ae. aegypti* vectors.

## 1. Introduction

**Figure 1:**
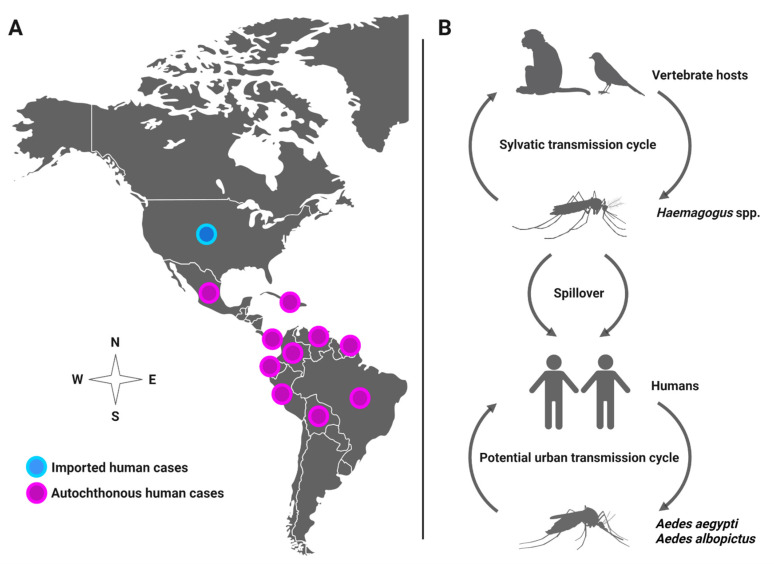
(**A**) Distribution of MAYV in the Americas. (**B**) Potential transmission cycles of MAYV.

Mayaro virus (MAYV) is a positive-sense single-stranded RNA virus in the *Alphavirus* genus that spreads among humans by the bite of infected mosquitoes. In most cases, human infection causes influenza-like illness (3–5-day duration) or may lead to severe debilitating arthralgia that persists for months [1,2,3]. MAYV has been detected through virus isolation or antibodies in several countries, such as Brazil, Venezuela, Ecuador, Peru, Panama, Mexico, French Guiana, and Colombia [4,5]. Recently, MAYV was recovered from a child with febrile illness in Port-au-Prince, Haiti, suggesting spread to the Caribbean region [6]. The patient was in proximity to a large urban environment and coinfected with dengue virus (DENV), which lends support to the likely role of *Aedes aegypti* as a MAYV vector. Although MAYV distribution is currently limited to Latin America, it has the potential to rapidly expand its geographical distribution and emerge in new regions in North America due to human movement and vector invasion, leading to local transmission, as observed with other emergent viruses, such as chikungunya (CHIKV) and Zika (ZIKV) viruses. For instance, studies have documented MAYV spreading to the U.S. and Europe from travelers infected with MAYV in French Guiana [7,8], Brazil [9], Peru [10], Ecuador [11], Bolivia [11], and Suriname [12], underscoring the potential for emergence in new regions (Figure 1A). Although MAYV is maintained in nature between forest-dwelling *Haemagogus* mosquitoes and non-human primates and perhaps other vertebrates (e.g., birds) in a sylvatic, enzootic transmission cycle, the anthropophilic mosquito *Ae. aegypti* is competent to transmit MAYV, suggesting potential urban transmission among human reservoir hosts, which may lead to a MAYV outbreak [2,6,13,14,15,16] (Figure 1B).

Temperature is a key climatic driver that can determine the risk of mosquito-borne pathogens. Changes in temperature can influence viral replication and dissemination within mosquitoes, which may modify the extrinsic incubation period, a sensitive parameter in the index of vectorial capacity [17]. The durations of the extrinsic incubation period of emerging viruses, including ZIKV [18], CHIKV [19], and DENV [20], are temperature-dependent and often observed to be shortened under elevated temperatures. Many studies have associated higher temperatures (>26 °C) with enhanced mosquito vector competence [21,22,23]; however, alternative outcomes (i.e., lower vector competence) were also observed under high temperatures [24,25,26,27]. The outcome of infection and associated vector competence across temperature typically follows a thermal performance curve with an optimal temperature(s) for infection and lower values above and below this optimal temperature [28]. These observations, together with predicted climate change, highlight the importance of considering temperature variation in the assessment of infection barriers in *Ae. aegypti* and transmission risk of MAYV. However, the influence of temperatures on infection, replication, and the extrinsic incubation period is not well described for MAYV. In this study, we characterize infection and growth kinetics of MAYV following per os infection in cohorts of *Ae. aegypti* at high and low adult-holding temperatures of 30 °C and 20 °C, respectively, for several time points under laboratory conditions. Determination of the impacts of temperatures on MAYV infection processes inside mosquito vectors is required to improve our understanding of how different temperature regimes may alter the emergence and epidemiology of MAYV.

## 2. Materials and Methods

### 2.1. Mosquitoes

Mosquitoes used to establish an *Ae. aegypti* colony were from larval collections made in 2020 from man-made containers located in Vero Beach, FL. Larvae were fed on 0.2 g of an equal mixture of lactalbumin and *Saccharomyces cerevisiae* yeast until pupation [29,30,31]. Newly emerged adults were maintained in a climate-controlled room with access to 10% sucrose at 28 °C and 60–70% humidity, with a 12 h/12 h light/dark cycle. Female mosquitoes were allowed to feed for 1 h on restrained chickens to initiate egg development. The blood-feeding process was carried out according to an established Animal Use Protocol (202007682). This protocol was approved by the University of Florida’s Institute of Animal Care and Use Committee. F5-generation mosquitoes from field-collected parents were used in the virus infection study.

### 2.2. Primate Cells

Non-human primate (Vero) cells (American Type Culture Collection, Manassas, VA, USA) were propagated on corning cell culture flasks (175 cm^2^) and kept in Medium 199 (HyClone, GE Healthcare, Logan, UT, USA) plus 10% heat-inactivated fetal bovine serum (Thermo Fisher Scientific, Waltham, MA, USA), antibiotics (penicillin–streptomycin), and Mycostatin. Primate cells were grown in a 5% CO_2_ atmosphere at 37 °C.

### 2.3. Infectious Bloodmeal Preparation

The prototype strain of MAYV (TRVL 4675, GenBank: MK070492.1) used in this study was originally isolated from a MAYV-infected human in Trinidad in 1954 and was obtained from the Center for Disease Control and Prevention branch in Fort Collins, CO, USA. Monolayers of Vero cells were inoculated with a dilute stock of MAYV and incubated for two days at 37 °C with 5% CO_2_. Following the two-day incubation, MAYV-infected cells and media were harvested and combined with defibrinated bovine blood (Hemostat, Dixon, CA, USA) and adenosine-5′-triphosphate disodium salt trihydrate (ATP, Thermo Fisher Scientific, Waltham, MA, USA) to prepare viremic bloodmeals for mosquito MAYV infection trials [32,33].

### 2.4. Mosquito per os Infection with MAYV

**Figure 2:**
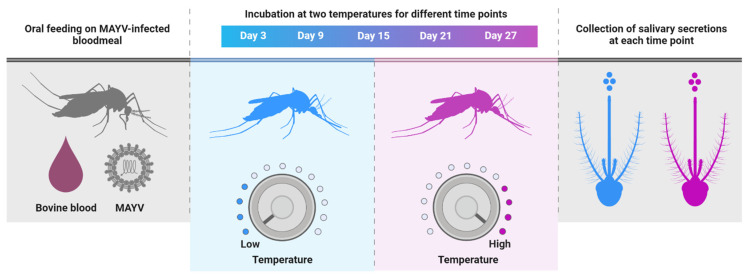
Schematic diagram of the experimental design.

For per os infection, four- to seven-day-old adult mosquitoes were allowed to ingest infectious bloodmeals containing MAYV (7 log_10_ plaque-forming unit equivalents (PFUe)/mL) delivered through a Hemotek membrane feeding system (Discovery Workshops, Lancashire, UK) preheated to 37 °C for 1 h at 28 °C. Prior to blood feeding trials, mosquitoes were deprived of sucrose for 24 h. After feeding, mosquitoes were anesthetized with CO_2_, and engorged mosquitoes were placed in new cages, held under two constant temperatures (30 °C or 20 °C), and provided with 10% sucrose (Figure 2). We deliberately chose two temperatures that approximate the average daily maximum and minimum temperatures in Florida during the summer months, which are associated with high risk for arbovirus transmission by *Ae. aegypti* [34]. Cohorts of mosquitoes (*n* = 30) were sampled at 3, 9, 15, 21, and 27 days post ingestion (dpi) of MAYV-infected blood. During each sample period, mosquitoes were anesthetized with CO_2_ and dissected to separate the body section from appendages (wings and legs) of individual mosquitoes. Tests for the presence of MAYV RNA in the bodies, legs, and salivary secretions were used as indicators of susceptibility to infection, disseminated infection, and transmission, respectively. Salivary secretions were collected using microhematocrit capillary tubes as previously reported [32]. Briefly, proboscises of mosquitoes were inserted into 1 mm microhematocrit capillary tubes (Thermo Fisher Scientific, Waltham, MA, USA) containing non-drying immersion oil (Cargille Laboratories, Cedar Grove, NJ, USA) for 45 min. All mosquito tissues (body and legs) were placed separately in individual microcentrifuge tubes containing 1 mL incomplete Medium 199 and stored at −80 °C until processing. Because salivary secretions were anticipated to have lower viral titers than other tested tissues, we combined them into a smaller volume of media (300 µL) to improve sensitivity. MAYV infection studies were carried out in accordance with the guidelines approved by the University of Florida’s Institutional Biosafety Committee and Biohazard Project Registrations.

### 2.5. Detection and Titration of MAYV

Mosquito tissues (bodies and legs) were homogenized for 3 min at 19.5 Hz using a TissueLyser II sample disruptor (Qiagen, Germantown, MD, USA), followed by centrifugation for sample clarification. MAYV RNA was extracted from 140µL of mosquito tissues and salivary secretions using a QIAamp viral RNA mini kit (Qiagen, Valencia, CA, USA) and eluted in 60 μL of buffer according to the manufacturer’s protocol. MAYV RNA was detected using Superscript III One-Step qRT-PCR with a Platinum Taq kit (Invitrogen, Carlsbad, CA, USA). Primers and probes for qRT-PCR were designed by Integrated DNA Technologies (Coralville, IA, USA) to target a nonfunctional structural polyprotein precursor gene and consisted of the following sequences: forward primer (5′-TGGACCTTTGGCTCTTCTTATC-3′), reverse primer (5′-GACGCTCACTGCGACTAAA-3′), and probe (5′-/56 FAM/TACTTTCCTGCTGCAAGGGCTCTT/3BHQ_1/-3). The run profile of the CFX 96 real-time PCR detection system (Bio-Rad Laboratories, Hercules, CA, USA) was as follows: 50 °C for 30 min, 94 °C for 2 min, 39 cycles of 94 °C for 10 s, 60 °C for 1 min, and 50 °C for 30 s. Titers of MAYV in mosquito tissues and salivary secretions were expressed as plaque-forming unit equivalents per mL (PFUe/mL). We estimated PFU equivalents (PFUe) for each MAYV-positive sample via regression analysis between PFU and quantification cycle (*C_q_*) values of MAYV stock viruses using the methods described in [35]. The plaque assay used in the standardizing method involved inoculating monolayers of Vero cells in six-well plates with 140 µL of serial dilution MAYV (3-fold replication per dilution), followed by a 1 h incubation at 37 °C in a 5% CO_2_ atmosphere. Following incubation, each well received a 2 mL agarose overlay (0.7%) and was incubated for two additional days. After incubation, media and agarose were removed, the plates were stained with crystal violet and rinsed with tap water, and visual plaques were counted. Each plaque is assumed to have originated from a single virus infection. MAYV infection was defined as the percent of mosquitoes with a MAYV-infected body among engorged mosquitoes. Disseminated infection and transmission were defined as the percent of mosquitoes with MAYV-infected legs and salivary secretions, respectively, among engorged mosquitoes.

### 2.6. Statistical Analysis

Infection, disseminated infection, and transmission rates were compared among temperature treatments and time points by logistic regression analysis (SAS Institute Inc., Cary, NC, USA). Comparisons of MAYV titers between treatments, as an indicator of growth kinetics, were analyzed using two-way ANOVA. *p*-values < 0.05 were considered statistically significant.

## 3. Results

**Figure 3:**
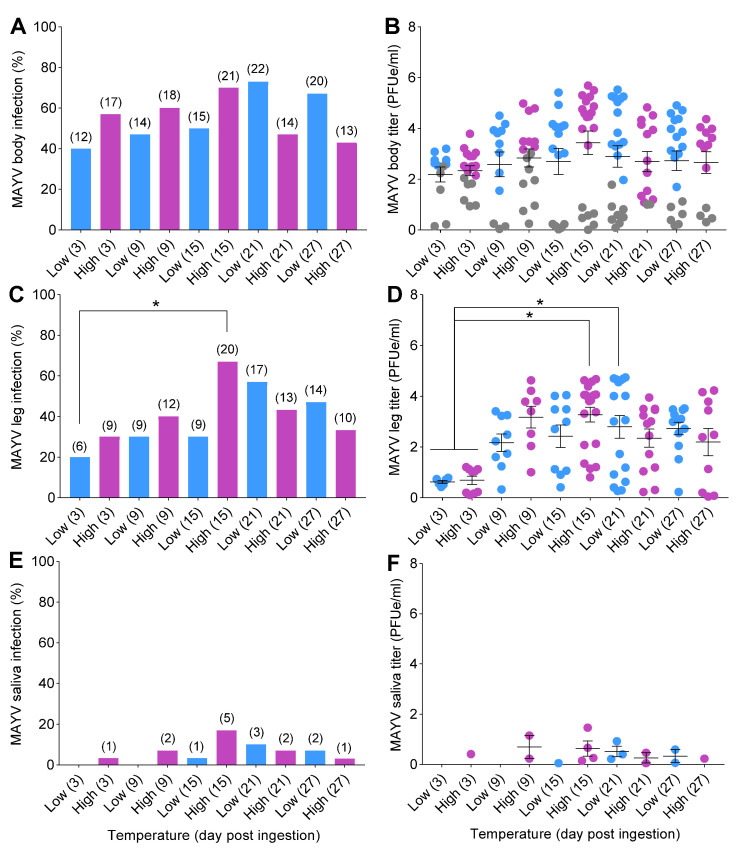
Temperature influences on mosquito competence for MAYV. Mosquitoes were challenged with ingestion of MAYV-infected bloodmeal (7 log_10_ PFUe/mL) for 1 h at 28 °C; then, engorged females were placed in separate environmental incubators (20 °C = low; 30 °C = high) for the remainder of the study. At 3, 9, 15, 21, and 27 dpi, mosquitoes (*n* = 30/time point) were anesthetized with CO_2_, and their wings and legs were removed from their bodies, followed by a collection of salivary secretions. (**A**) MAYV susceptibility to infection (body), (**C**) disseminated infection (legs), and (**E**) transmission (salivary secretions) expressed in percentages. The kinetics of MAYV growth in mosquito tissues and salivary secretions were determined by qRT-PCR. (**B**) MAYV titer of body, (**D**) legs, and (**F**) salivary secretions, represented as (PFUe/mL). Each data point in (**B**,**D**,**F**) represents the kinetics of MAYV growth (titer) of individual mosquitoes. Gray data points in (**B**) represent mosquitoes with non-disseminated infection (i.e., MAYV infection limited to the midgut). Numbers in brackets above bars indicate the total number of mosquitoes positive for MAYV. Horizontal lines indicate the mean ± SEM. Statistical significance was determined using logistic regression analysis and two-way ANOVA for MAYV infection measurements and titers, respectively. *p*-values were corrected for multiple comparisons. Asterisks (*) denote significant differences; *p* < 0.05.

Mosquitoes were exposed to infectious bloodmeals containing MAYV, and a total of 300 fully engorged females were assayed for infection, disseminated infection, and transmission at 3, 9, 15, 21, and 27 dpi under two temperature conditions. The status of MAYV infection in mosquitoes was confirmed by qRT-PCR. Logistic regression detected a significant interaction effect between temperature and time (Table 1) on MAYV susceptibility to infection. After correcting *p*-values for multiple comparisons, we did not detect differences in susceptibility to infection between the two temperatures across time (Figure 3A). For disseminated infection (spread of MAYV beyond the midgut into the hemocoel), we found a significant effect of time (Table 1). There were no effects of temperature, time, or their interaction on MAYV transmission (Table 1). 

There were trends of increases in infection and disseminated infection rates over time, followed by reduction at later time points, in mosquitoes under both temperature conditions (Figure 3A,C). Infection and disseminated infection rates were higher at early time points (3, 9, and 15 dpi) for mosquitoes incubated at high temperature in comparison to those incubated at low temperature. Peaks of infection and disseminated infection in mosquitoes were observed on day 15 at 30 °C and on day 21 at 20 °C (Figure 3A,C). Transmission was first observed on day 3 for the constant 30 °C treatment but not until day 15 for mosquitoes held at 20 °C (Figure 3E). Low transmission rates (<25%) attributable to a salivary gland barrier precluded the calculation of the extrinsic incubation period (EIP_50_) for cohorts of mosquitoes (Figure 3E,F).

Two-way ANOVA analyses demonstrated no significant effects of treatment on MAYV titers in mosquito bodies (*F* = 0.96; *df* = 9,20; *p* = 0.49) or salivary secretions (*F* = 0.83; *df* = 9,20; *p* = 0.59) (Table 2). However, we detected a highly significant effect of time on MAYV titers in legs of mosquitoes (*F* = 4.49; *df* = 9,20; *p* = 0.002) (Table 2). The titers in mosquito bodies and legs tended to be higher 3, 9, and 15 dpi at 30 °C than at 20 °C (Figure 3B,D).

## 4. Discussion

Temperature variation is an important driver that shapes mosquito and virus interactions and subsequent transmission to hosts. Here, we measured susceptibility to infection, disseminated infection, and transmission patterns of MAYV in *Ae. aegypti* at multiple intervals during infection. We used two temperatures, which approximate the average daily maximum and minimum temperatures that mosquitoes encounter during the summer months in Florida, when the risk for arbovirus transmission is the highest. Our data demonstrate that although rates of MAYV susceptibility to infection and disseminated infection tended to be higher in percentage at 30 °C than 20 °C at earlier time points during infection, infection measures were lower at 30 °C than at 20 °C at later time points during infection. Results also show that the MAYV replication kinetics were relatively greater at early time points in mosquitoes held at 30 °C than those held at 20 °C. Although temperature alone did not alter infection rates, the interaction between temperature and time indicates that mosquitoes held at warmer extrinsic incubation temperatures may facilitate earlier midgut escape and dissemination of MAYV and increase the number of mosquitoes exhibiting a disseminated infection than at cooler temperatures. High temperature may shorten the extrinsic incubation period of MAYV, enhancing transmission potential. However, evidence of transmission early in the infection (day 3) is based on a single positive mosquito out of 30 individuals tested, so results should be interpreted with caution. 

In our study, we found little evidence that elevated incubation temperature within the tested range increased MAYV infection and disseminated infection rates. However, we did observe that higher temperature allowed transmission to occur as early as day 3, whereas transmission did not occur until day 15 in mosquitoes held at a cooler temperature. This observation suggests that high temperatures may enhance viral replication, midgut escape, and dissemination to secondary tissues (e.g., body fat, hemocytes, and nerve tissue), enabling MAYV to rapidly infect salivary glands. Our observations follow similar patterns to those of other studies that demonstrated alterations in the progression of viral infection and extrinsic incubation periods under different holding temperatures in several virus vector systems [17,36,37]. For instance, mosquitoes held at high temperatures were associated with higher rates of infection and transmission and shorter extrinsic incubation periods for ZIKV and *Ae. aegypti* [18], DENV and *Ae. albopictus* [20,23], West Nile virus and *Culex univittatus* [38], and Japanese encephalitis virus and *Cx. pipines* [39]. A transcriptome profile study of *Ae. aegypti* reared at different temperatures (20, 28, and 36 °C) characterized temperature-triggered transcriptional changes, aiding in defining molecular mechanisms linking changes in innate immunity genes to variation in environmental temperature [36]. Variation in temperature is predicted to influence insect immunity and interactions between vectors and pathogens [17,40]. For example, cool temperatures inhibit the RNA interference pathways essential to antiviral immunity in mosquitoes and *Ae. aegypti* infection with CHIKV and yellow fever virus [41]. Along the same lines, changes in temperature have been linked to changes in expression of stress genes and immune genes, such as those encoding antimicrobial peptides cecropin and defensin, as well as Sindbis virus (SINV) infection in *Ae. aegypti* [42]. 

We detected few differences according to comparisons of treatments, suggesting MAYV vector competency of Florida *Ae. aegypti* is robust over a range of environmental temperatures. Previous studies have reported higher MAYV infection and titers over time in *Ae. aegypti* [15,43,44,45]. At later time points, however, there were apparent progressive declines in viral infection, titer, and dissemination, suggesting a modulation of infection by mosquitoes at later stages of infection under both 30 °C and 20 °C temperatures. The level of viral modulation was more obvious in mosquitoes maintained at 30 °C than at 20 °C. Our observation is consistent with a previous report that showed that at an elevated temperature (32 °C), mosquitoes were able to better modulate an *Alphavirus* western equine encephalomyelitis virus [24,46]. Although the mechanism underlying viral modulation is unclear, it may be attributable to mosquitoes’ innate antiviral immune defenses, such as the RNA interference pathway, which has been shown to limit CHIKV, DENV, and SINV replication in *Ae. aegypti* [47,48,49,50]. Silencing of the expression of protein AGO2, an essential component of the small interfering RNA pathway (siRNA), controlled MAYV replication in *Ae. aegypti* from Brazil [51]. 

Florida *Ae. aegypti* mosquitoes have been shown to be efficiently competent to transmit major viruses of public health importance (e.g., ZIKV [52], CHIKV [53,54], and DENV [35]). However, earlier reports demonstrated that *Ae. aegypti* is a poor vector for MAYV [14,15]. In agreement with these reports, we observed low rates of salivary secretion infection in *Ae. aegypti* under all treatment conditions. Our findings, along with those reported in [15], suggest that despite modest rates of disseminated infection, a substantial salivary gland barrier in Florida *Ae. aegypti* may limit its potential as a vector of MAYV in this state. In contrast, progeny of *Ae. aegypti* collected from the city of Belo Horizonte, Brazil, showed much higher saliva infection (69.5%) using the same Trinidad strain of MAYV as that used in the current study [45]. Observed differences in vector competence within species could be due to the genetic background or microbiota of the vector, which can vary with geographical origin [55,56,57]. However, the viral titers in culture medium were 100-fold higher than in those obtained in current study, which likely contributed to this observation. Along the same lines, a Brazilian field population of *Ae. aegypti* was permissible to MAYV, and high viral prevalence was observed in saliva after ingesting 10^8^–10^9^ PFU/mL infected blood and following incubation [58]. Natural infection of MAYV has been reported in both *Ae. aegypti* and *Cx. quinquefasciatus* in the Cuiabá region of Brazil [59]. Mosquitoes, such as *Anopheles quadrimaculatus* and *Ae. albopictus* from New York, were found to be competent for MAYV transmission based on laboratory infection experiments, with *Ae. albopictus* exhibiting higher infection rates [60]. Taken together, these observations suggest the need to determine the competence of mosquito species from different geographical origins to transmit MAYV with standardized methods [61], as well as monitoring of scenarios of climate change and entomological surveillance in the epidemiology of this virus. 

## 5. Conclusions

We characterized the progression of infection and replication of MAYV in *Ae. aegypti* within the range of temperature during the summer months associated with high risk for arbovirus transmission by this mosquito vector. Transmission of MAYV, as measured by the detection of viral RNA in salivary secretions, was delayed under cooling conditions compared to warmer conditions. Although *Ae. aegypti* mosquitoes were highly susceptible to MAYV infection, in our study, low saliva infection was found to possibly decrease risk of MAYV transmission in Florida. 

## Figures and Tables

**Table 1:** Logistic regression analyses for the effects of temperature, time, and their interaction on *Ae. aegypti* responses to infection (body), disseminated infection (legs), and transmission (salivary secretions) for MAYV. The temperature–time interaction is represented by temperature*time. Values in boldface indicate that the effect was significant (*p* < 0.05).

Mosquito Sample	Factor	*df*	*χ2*	*p*
Body	Temperature	1	0.001	0.97
	Time	4	2.33	0.67
	Temperature*time	4	11.92	**0.017**
Legs	Temperature	1	0.96	0.32
	Time	4	10.12	**0.03**
	Temperature*time	4	8.63	0.07
Salivary secretions	Temperature	1	1.06	0.30
	Time	4	2.82	0.58
	Temperature*time	4	3.02	0.55

**Table 2:** Two-way ANOVA for the effects of temperature, time, and their interaction on MAYV titers in *Ae. aegypti* tissues and salivary secretions. The temperature–time interaction is represented by temperature*time. Values in boldface indicate that the effect was significant (*p* < 0.05).

Mosquito Sample	Factor	*df*	*F*	*p*
Body	Temperature	1	0.07	0.79
	Time	4	1.27	0.31
	Temperature*time	4	0.89	0.48
Legs	Temperature	1	0.15	0.69
	Time	4	9.55	**0.0002**
	Temperature*time	4	0.52	0.72
Salivary secretions	Temperature	1	0.21	0.64
	Time	4	0.35	0.84
	Temperature*time	4	1.47	0.24

## Data Availability

The data presented in this study are available on request from the corresponding author.
